# Quantitative evaluation of a theoretical-conceptual model based on affective and socio-behavioral dimensions to explain the academic performance of mathematics students

**DOI:** 10.3389/fpsyg.2024.1372427

**Published:** 2024-08-07

**Authors:** Felipe Marín-Álvarez, Luis Flores-Prado, Oriana Figueroa, Pablo Polo, Jorge J. Varela, José Antonio Muñoz-Reyes

**Affiliations:** ^1^Programa de Doctorado en Educación, Vicerrectoría Académica, Universidad Metropolitana de Ciencias de la Educación, Santiago, Chile; ^2^Instituto de Entomología, Universidad Metropolitana de Ciencias de la Educación, Santiago, Chile; ^3^Laboratorio de Comportamiento Animal y Humano, Centro de Investigación en Complejidad Social, Facultad de Gobierno, Universidad del Desarrollo, Santiago, Chile; ^4^Instituto de Bienestar Socioemocional, Facultad de Psicología, Universidad del Desarrollo, Santiago, Chile

**Keywords:** education and development, sociability, social interaction, human behavior, educational mathematics, educational psychology

## Abstract

**Objective:**

There is evidence that suggests that affective dimensions, personality traits, as well as students’ cooperative interpersonal interactions, are an important element in the students learning process. In this work we propose a theoretical model, based on evidence, that shows the direct and indirect relationships between these factors and academic performance in mathematics courses, in undergraduate and school students.

**Methods:**

To understand the type of relationships between these variables, the PANAS psychometric test of positive and negative affect, the BIG FIVE personality test and the economic decision game DUPLES GAME were applied. The study sample was 130 students between 17 and 22 years of age from undergraduate and school (*M* ± *SD* = 20.1 ± 3.99).

**Results:**

From a path analysis, statistically significant relationships were found, for example, a direct relationship between neuroticism and positive affect, which in turn is related to academic performance. We also found a direct relationship between neuroticism and negative affect, extraversion and positive affect. This allows us to propose that some of the independent variables of the model directly and indirectly influence the academic performance of students in the subject of mathematics.

**Conclusion:**

Positive affect and negative affect directly affect academic performance in mathematics, neuroticism has a direct impact on negative affect and extraversion direct impact on positive affect. Consequently, there are direct and indirect relationships between personality traits and affective dimensions, which affect the academic performance of mathematics students.

## Introduction

In the teaching of mathematics in school and undergraduate contexts, manifestations of aversion and unpleasant memories have been seen in students (Orjuela et al., 2019). There are students who say they feel intimidated (Maninat, 2021), generating massive copies, impersonations, and bad practices (Goldin et al., 2016).

Furthermore, there is evidence that suggests different associations between the learning obtained, reflected in academic performance, which is influenced by affective dimensions of students, their personality traits and the interactions that occur among them in educational contexts. Regarding affective dimensions, positive affect reflects the point to which a person feels active, alert, and energetic (Watson et al., 1988), anticipating rewarding experiences in those who experience them (Sánchez et al., 2008), which favors creativity and motivation (Schmidt, 2008). As well as, negative affect represents a general dimension of distress and unpleasant involvement, including a variety of aversive emotional states such as anger and fear (Watson et al., 1988). In this line, a relationship between affective dimensions and mathematics learning has been demonstrated, for example, in front of an exercise, where students can connect with the emotional experience they have had previously (Gómez, 2016). Thus, when a higher level of abstraction is required, as in algebraic factorizations or calculus in general, triggers of negative emotions have been evidenced (Dorier, 1991; Cerda et al., 2016), where the most frequent states are frustration and confusion, marked by boredom and anxiety (Di Leo et al., 2019).

Regarding mathematics anxiety, it is inversely related to results on mathematics tests (Hembree, 1990), presenting a significant impact on academic performance (Caviola et al., 2021). Furthermore, this inverse relationship between mathematics anxiety and academic performance begins in childhood and remains significant until adulthood (Barroso et al., 2021). There is even a special behavior because poor performance in mathematics can cause mathematics anxiety and, at the same time, mathematics anxiety reduces academic performance (Carey et al., 2016). Along these lines, it is necessary to specify that academic performance in mathematics in Chile corresponds to the arithmetic average calculated with all summative evaluations, while general performance is calculated with all subjects. Specifically in algebra, these difficulties are produced by a higher complexity due to the epistemological nature of these subjects, characterized by the requirement of many new and abstract concepts, together with an absence of graphical examples or geometric associations that facilitate their understanding (Oktaç and Gaisman, 2010; Costa and Rossignoli, 2017). Furthermore, there is a positive effect when there is a good predisposition towards the development of mathematical exercises which in turn reduces anxiety towards learning these elements (Akin and Kurbanoglu, 2011). Thus, the enjoyment of mathematics presents (mild) positive effects on subsequent perceived effort and beliefs about mathematical competence (Pinxten et al., 2014). Likewise, beliefs about mathematical competence have positive effects on mathematical performance and negative effects on the perceived expenditure on mathematical effort (Pinxten et al., 2014).

Consequently, due to the existence of mathematical contents that present greater and lesser difficulty to understand and assimilate (Castro et al., 2020), negative affect is associated with a lower level of achievement and positive affect with a higher one (Pekrun et al., 2017). Additionally, there are factors that contribute to predict academic performance such as those that reflect the development of cognitive abilities together with those that refer to individual characteristics such as personality traits (Ackerman and Beier, 2006). Personality traits correspond to attributes that show associations with important activities and phenomena on an individual and social scale, such as work and school performance (John et al., 1994). Therefore, personality traits allow predicting behaviors of various types such as academic performance (Cupani et al., 2013) even being statistically significant predictors (O’Connor and Paunonen, 2007), so that personality traits have an influence on students’ academic performance (Rothstein et al., 1994). Regarding extraversion, a personality trait characterized by a high display of activity, sociality, and expressiveness (Benet and John, 1998; Diener et al., 2003), there is evidence that shows both a positive and a negative relationship with academic performance.

There are studies that show that more extroverted students have lower academic performance (Goff and Ackerman, 1992; Bauer and Liang, 2003; Furnham et al., 2003), which could be explained by differences in the study time spent by students with different levels of extraversion. In this regard, one study showed that less extroverted students spend more time studying, while more extroverted students spend more time socializing (Chamorro and Furnham, 2005). In contrast, Rothstein et al. (1994) reported that extraversion is positively correlated with students’ class participation which would generate a positive attitude towards learning (De Raad and Schouwenburg, 1996). Specifically, extroverted students differ significantly from introverted students in higher performance on summative assessments involving exercises in which logical reasoning is required (Furnham et al., 1998). Regarding agreeableness, Furnham and Chamorro (2004) suggest that it is a personality trait that would not be associated with academic performance. However, positive relationships with academic performance have been found (Farsides and Woodfield, 2003; Conard, 2006) as well as negative ones (Rothstein et al., 1994; Paunonen, 1998). Given the above, the favorable or unfavorable relationships of this personality trait with respect to performance are not yet entirely conclusive. As for conscientiousness, the explanatory power of this trait is due to the motivational properties reflected in effort and persistence (Chamorro and Furnham, 2005). That is, it is likely that those students who are more organized and self-disciplined achieve better academic results compared to those who do not possess these characteristics (Cupani et al., 2013).

Neuroticism, a personality trait referred to emotional instability and excessive worry with high degrees of anxiety, is associated with cognitive abilities (Eysenck, 1967), as well as with self-efficacy beliefs, a predictive variable of academic performance, so the effect of this personality trait could be indirect and mediated by other variables (Judge and Ilies, 2002). Finally, openness to experience, a personality trait that has been interpreted in terms of capacity (Johnson, 1994; Chamorro and Furnham, 2005), is characterized by creativity, originality, and exploration of the unknown, which would allow inferring a relationship with the levels of achievement of the activities proposed (Ackerman and Heggestad, 1997) and with academic performance in children and adolescents independent of intelligence (Cupani et al., 2013; Poropat, 2014; Zhang and Ziegler, 2015). Thus, the importance of these elements is because the influence of personality traits on academic performance in various subjects, such as mathematics, have been demonstrated (Spinath et al., 2010; Zhang and Ziegler, 2015). Thereby, extraversion presents a certain correspondence with positive affect and neuroticism with negative affect (Watson and Pennebaker, 1989; Clark et al., 1994), where neuroticism is a personality trait reflecting low stability and high emotional dysregulation (Benet and John, 1998; Del Valle et al., 2020), which is associated with a tendency to experience negative emotions (Matsumoto, 2006; Nettle, 2006; Austin et al., 2008; Reisenzein and Weber, 2009; Andrés et al., 2014; Pollock et al., 2016). Considering this evidence, interactions between affective components and some personality traits of individuals are visualized.

Cooperation among students is of interest in mathematics learning because peer interactions occur in a classroom, where collaborative problem-solving fosters social interaction and is positively related to academic outcomes (Tudge et al., 1996). This occurs, for example, when a student with lower levels of academic achievement is offered active participation, with explanations and stimuli elaborated by a higher-achieving peer (Fuchs et al., 1994). These interactions can facilitate engagement and the development of intellectual skills toward study because of the option to coach higher achievers in cooperative strategies (Fuchs et al., 1994). Thus, active classrooms have fostered greater interaction among students over a traditional lecture-based format (Pulgar et al., 2020). This interaction has allowed observing a significant association of social relationships among peers with academic performance (Candia et al., 2022; Pulgar et al., 2022). Accordingly, a significant association between students’ social relationships and their academic performance at different ages is highlighted in the literature (Baldwin et al., 1997; Caprara et al., 2000; Bruun and Brewe, 2013; Ivaniushina and Alexandrov, 2018; Berthelon et al., 2019; Candia et al., 2019; Pulgar et al., 2020). Complementarily, it is possible to note a link between some personality traits and behavioral attributes that are part of the cooperative behavior evidenced by students in different contexts. Conscientiousness is a personality trait characterized by impulse control and task-oriented and goal-oriented behavior (Benet and John, 1998). It is thought that students with high conscientiousness are more organized and present greater motivation for good academic results (Martínez De Ibarreta et al., 2011). Considering this background, it is possible to infer that students with a high level of this personality trait present low levels of cooperation with other students who have minimal levels of conscientiousness, when facing academic challenges. For example, Komarraju and Karau (2005) studied personality traits in relation to academic performance and found that students with higher conscientiousness scores presented greater competence, and therefore less cooperation, with their peers. Regarding agreeableness, Tobin et al. (2000) indicate that people with a more marked presence of this trait control their negative affect and make more effort to please others. Alternatively, agreeableness is a personality trait that includes characteristics such as altruism, in addition to a prosocial and community orientation towards others (John et al., 2011), so it could be linked to cooperative-type interpersonal interactions among students. Furthermore, personality traits have been studied in children and adolescents from third-party evaluations (Digman and Takemoto-Chock, 1981; John et al., 1994) but with little evidence from measures in people with an age group between ages 15 and 19 (Romero et al., 2002).

For educational mathematics, the study of the affectivity present in mathematics has been a subject of research since the seventies. Becker (1983) provides an overview of emotional reactions to mathematics which has led to the emergence of theoretical models such as the one proposed by Buxton (1981) in his book ‘Do you panic about mathematics?’ With this background, McLeod (1988) integrates the emotional responses of students working with routine mathematical problems, which were considered important motivating forces due to positive or negative reactions that could arise. These works in the field of educational mathematics made it possible to “build an approach to affect in a theoretical framework based on cognitive science” (McLeod, 1994, p. 642). However, these studies paid little attention to affect and the types of personality traits and interactions in the classroom, which establishes the need to develop a more integrated approach to the investigation of affectivity and other dimensions of personality and behavior in the classroom. Mathematics, theoretical elements that have served us for the construction of the theoretical-conceptual model that we propose here.

### Current study

The previous antecedents show the incidence of affectivity, personality, and socio-behavioral traits in the learning of mathematics; but there is no analysis that explores the interrelationships between these variables and academic performance in mathematics. Thus, the aim of our study is to establish the link between academic performance in mathematics and affective dimensions, personality traits and cooperative behaviors, as well as the associations between these variables, in students in higher education and school education. In this way, knowledge is provided for future curricular innovations that incorporate these dimensions.

## Materials and methods

### Participants

Data were obtained from a sample of undergraduate students taking the subject “Mathematics 3” at the Universidad Andrés Bello, at the Santiago and Viña del Mar campuses, and third year students of Colegio San Adrián de Quilicura, both institutions located in Chile. This sample was formed due to the failure and dropout rates that existed in both institutions, along with comments rejecting the subject. Furthermore, certain stability has been seen in personality traits in young people of these ages (Abella and Bárcena, 2014), which is why these students of these ages were chosen.

Thus, they were chosen at random three courses of students of the Commercial Engineering career at Universidad Andrés Bello, in which the subject “Mathematics 3” was taught, due to the fact that students frequently recognize a high level of difficulty in passing this subject (personal communication), which is also corroborated with the existing failure rate (e.g., 39% in 2016; 37% in 2017; 42% in 2018; 27% in 2019). Furthermore, based on what the school authorities reported (personal communication), based on complicated and difficult content, such as basic elements of trigonometry, as well as arithmetic work with functions, a third-year course was chosen at random. San Adrián School. This course teaches elementary mathematical content required for the aforementioned university subject. For this reason, there are curricular similarities between school and university mathematics content, in addition to there being no considerable difference in age, reasons that justify the selection of these study groups. The sample consists of 130 students (75.3% male) with M±SD=20.1±3.99 , as shown in Table 1.

**Table 1:** Number of research participants.

	University	School	Total
Men	78	20	98
Women	26	6	32
Total	104	26	130

### Ethical authorization

The study was conducted in accordance with the Declaration of Helsinki. The protocol for data collection in students was approved by the Ethics Committee of the University of Santiago de Chile code 258, prior to the application of the instruments, for which informed consent and assent was also obtained from all participants and guardians of participants in this research. Data collection was carried out between August and October 2022, in university students, and during November of the same year in school students.

### Psychometric measurement of positive and negative affect

The Positive Affect and Negative Affect Schedule (PANAS) instrument was applied, widely used for recording emotions, which consists of a set of 20 words describing feelings and emotions, corresponding to the subdimensions Positive Affect (PA) and Negative Affect (NA), but this information is unknown to the respondents.

The version used of said instrument was adapted by Dufey and Fernandez (2012) for Chilean students, with the following levels of internal consistency measured by means of Cronbach’s alpha indicator: α=0.79 in men and α=0.73 in women, for the PA subdimension, and α=0.79 in men and α=0.83 in women, for the NA subdimension. These subdimensions present low correlations and no statistical significance, so that both scales are orthogonal in the assessment of affectivity (Dufey and Fernandez, 2012). Students were asked to read each of the words and choose an option according to how they generally feel, assigning a number from 1 to 5 according to the following detail: 1 = “very slightly or not at all,” 2 = “a little,” 3 = “moderately,” 4 = “quite a lot,” 5 = “extremely.”

### Psychometric measurement of personality traits

The Big Five personality measure (John et al., 2011), in its Spanish version (Benet and John, 1998), was also applied. The instrument consists of 44 items that assess dimensions defined on a 5-point Likert scale (1 = “strongly disagree”; 5 = “strongly agree”). Five personality factors are measured: Extraversion; Agreeableness; Conscientiousness; Neuroticism and Openness to Experience (Benet and John, 1998).

### Economic game

To measure the cooperation or cooperative tendency of the students, the “Duples Game” was implemented using an online interface through computers connected to the network (Candia et al., 2022). Student groups were formed with a maximum of 6 participants, where each of the members played with the rest of the group members, forming pairs for each round of the game. In each of these rounds, each student had 10 chips to distribute, where he had to decide how many to give to the person he was playing with and how many to keep (e.g., he could give all the chips and keep none, or give 2 chips and keep 8, etc.). At the end of the game, the amount of chips collected by each participant was converted to real Chilean money (each chip was equivalent to $50 Chilean pesos), therefore, the final profit of each participant depended on the amount of chips donated, not donated, and received.

### Measurement of academic performance

Subsequently, each student was given a summative evaluation of mathematics on a numerical scale with a decimal from 1 to 7, according to the educational level in which he/she was. Thus, the test applied in the university course was prepared by the teaching team that taught the course, while the test applied in the school course was prepared by the team of teachers belonging to the high school level.

**Figure 1:**
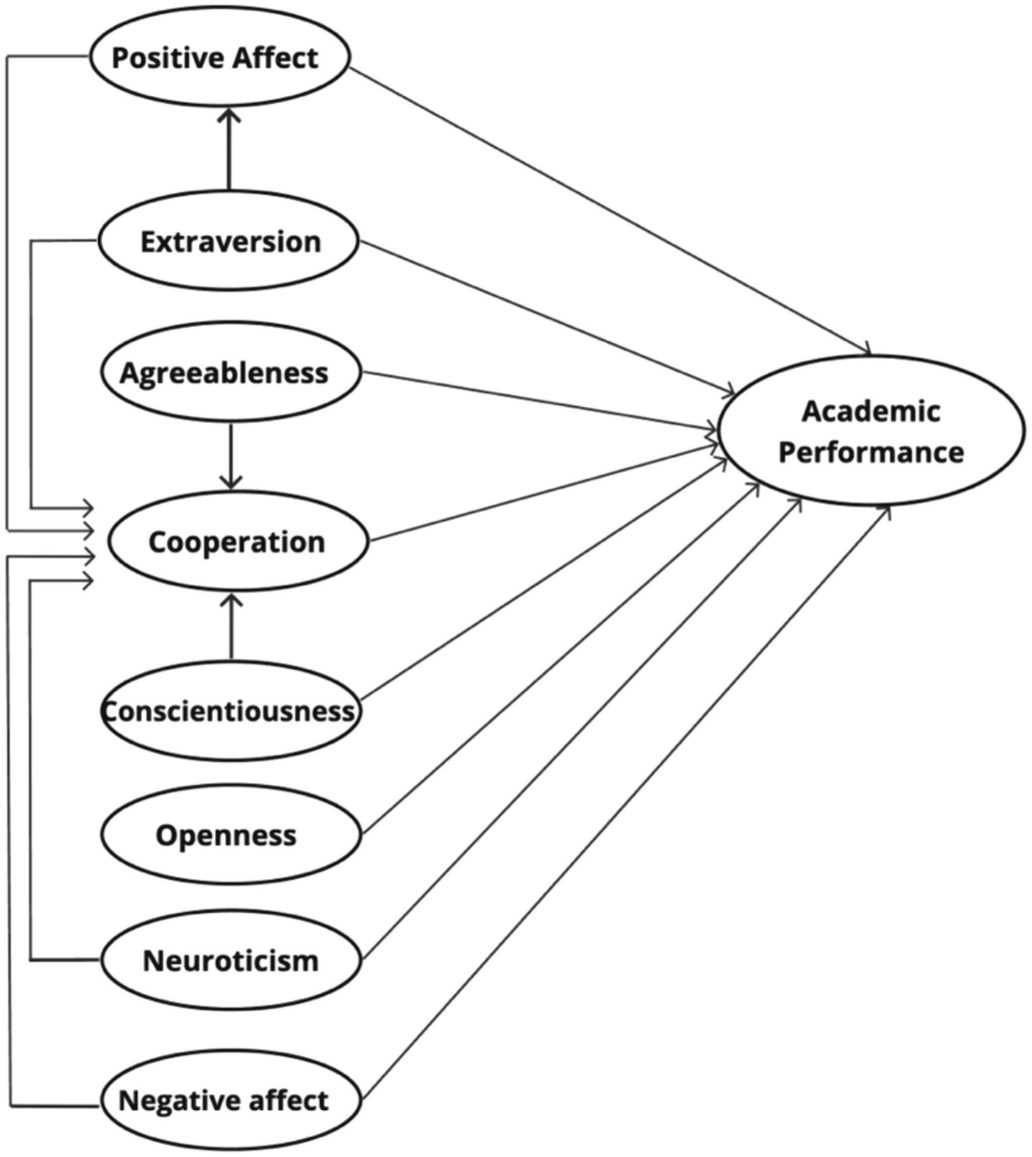
Direct and indirect relationships of variables.

Based on the literature review, a theoretical model was constructed that predicts direct and indirect relationships between independent variables and the academic performance obtained in a mathematics evaluation, as shown in Figure 1. This theoretical model serves as a basis for future replication studies, essential for the testing and verification of this proposed model.

### Statistical analysis

To test our predictions, a path analysis (Bollen and Curran, 2006) was performed to statistically evaluate the relationships between variables presented in the theoretical model (Figure 1) through structural equation modeling (Ruiz et al., 2010). SPSS Statistics 25 software (IBM) was used together with the AMOS extension. We considered the qualification obtained in the mathematics test as the dependent variable (academic performance) and the dimensions positive and negative affect, cooperation, and personality traits as independent variables. In addition, in the model we controlled for the establishment and the biological sex reported by the students. A significance level of α=0.05 was established.

## Results

**Figure 2:**
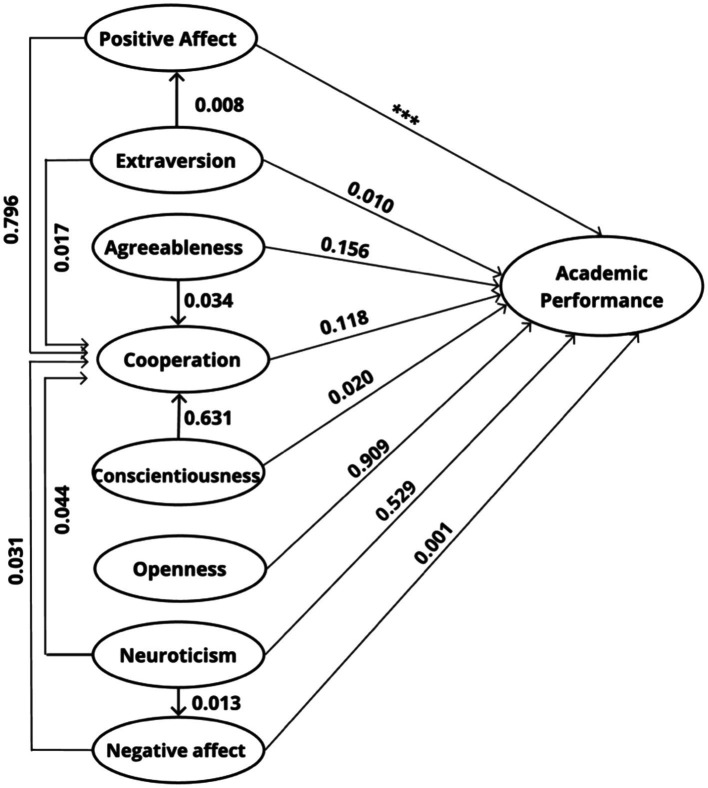
Statistical significance level of direct and indirect variables.

Different statistically significant relationships were found in the pre-established direction according to the theoretical model, as shown in Table 2 ( ∗∗∗p<0.001 ) and Figure 2. Also, we use an ML as a maximum likelihood estimation model. These relationships confirm the existence of variables that directly and indirectly affect academic performance in mathematics. The variables that show statistically significant direct relationships with mathematics performance are positive affect, extraversion, diligence, negative affect. In addition, the variables that mediate the impact on mathematics performance are extraversion and neuroticism. We did not find differences according to educational level in personality traits, affective dimensions, and cooperation. The model variables are the following: Extraversion ( M=3.35,SD=0.47,α=0.26 ), Agreeableness (*M* = 3.23, *SD* = 0.41, α = 0.33), Conscientiousness (*M* = 3.59, *SD* = 0.37, α = 0.22), Neuroticism (*M* = 3.22, *SD* = 0.41, α = 0.33), Openness (*M* = 3.48, *SD* = 0.47, α = 0.52), PA (*M* = 32.02, *SD* = 8.22, α = 0.84), NA (*M* = 26.34, *SD* = 9.61, α = 0.86), Cooperation ( M=4.95,SD=2.44 ).

**Table 2:** Level of significance of the relationships involved.

Relation	Estimate	S.E.	C.R.	P
Extraversion to PA	0.398	0.151	2.635	0.008
Neuroticism to NA	0.515	0.207	2.491	0.013
Extraversion to cooperation	−1.080	0.453	−2.386	0.017
Agreeableness to cooperation	1.074	0.506	2.121	0.034
PA to cooperation	−0.066	0.257	−0.258	0.796
Conscientiousness to cooperation	0.279	0.581	0.480	0.631
Neuroticism to cooperation	1.037	0.513	2.019	0.044
NA to cooperation	−0.460	0.213	−2.155	0.031
PA to academic performance	0.825	0.167	4.934	***
Extraversion to academic performance	−0.778	0.301	−2.586	0.010
Agreeableness to academic performance	−0.475	0.335	−1.418	0.156
Cooperation to academic performance	0.090	0.057	1.564	0.118
Conscientiousness to academic performance	0.881	0.378	2.328	0.020
Neuroticism to academic performance	−0.214	0.339	−0.629	0.529
NA to academic performance	−0.455	0.141	−3.220	0.001
Level to academic performance	1.575	0.334	4.721	***
Openness to academic performance	0.034	0.295	0.115	0.909

The academic level presents a positive and statistically significant relationship in academic performance in mathematics, which can be understood due to the accumulation of knowledge in students as they advance in their educational process. However, this variable has not been considered in the theoretical model because we only consider the affective variables, personality traits and cooperation, as seen in Figure 2. There is no data dependence or grouping within the sample, so we discard correlations between classes, that is, there is no serial autocorrelation between the residuals (Aguilar et al., 2012), Durbin-Watson statistic DW=1.87 . This is important in studies with SEM procedures where independence of the data is required.

**Figure 3:**
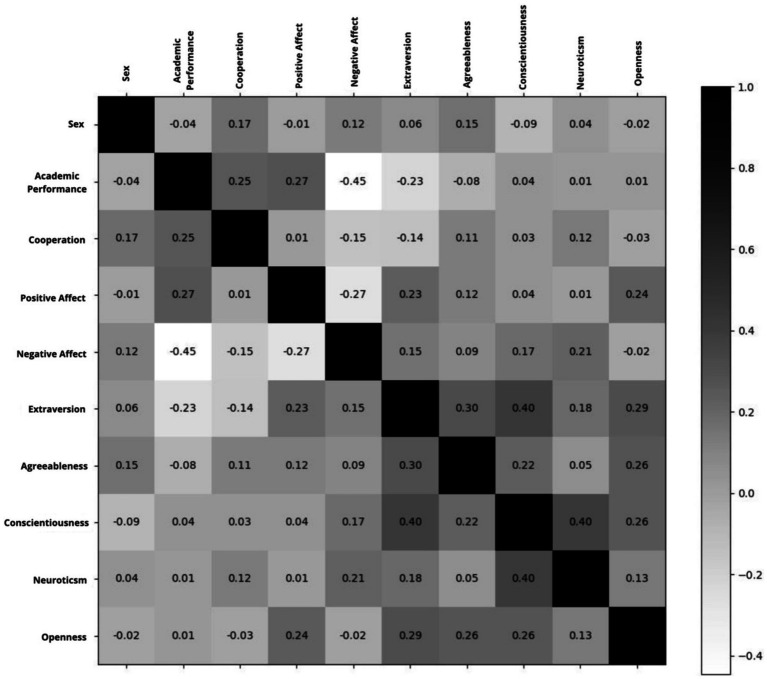
Correlation matrix.

We use the Kolmogórov-Smirnov test (K-S) and we find normal distribution in our variables Academic Performance ( p=0.218 ), PA ( p=0.591 ), NA ( p=0.237 ), Extraversion ( p=0.359 ), Agreeableness ( p=0.323 ), Conscientiousness ( p=0.200 ), Openness ( p=0.145 ), Neuroticism ( p=0.257 ). Also, we use the Levene test and we find that there is homogeneity of variance ( p=0.445 ). The correlations between the variables of the model are seen in the Figure 3.

## Discussion

From our results, the direct relationship of PA with academic performance, as well as the inverse relationship of NA with academic performance, coincides with the results obtained in another research (Watson et al., 1988; Sandín et al., 1999). During learning and exercising in mathematics, interactions between the affective and cognitive are developed (McLeod, 1994), which leads the student to connect with the affective experience he/she has presented in his/her academic journey (Gómez, 2016). NA is related to physical stress and anxiety symptoms (Toro et al., 2018) elements of importance in the process of teaching and learning mathematics. In the understanding of contents that require a higher level of abstraction such as algebra, polynomials or algebraic management of functions, negative emotions arise in students who in many cases experience mathematical anxiety (Klein et al., 2019), which supports the idea that mathematics is difficult to learn regardless of the student’s educational level (Castro et al., 2020). Regarding PA, its association with the occurrence of positive events in interpersonal relationships has been documented (Hamilton et al., 2017). This is relevant because it could generate a transit towards these emotions by controlling the effects of emotional dysregulation based on fear and anxiety (Klein et al., 2019), even more so when in PA there is a component of importance in the study of mathematics such as alertness (Watson et al., 1988).

Furthermore, we found significant relationships between positive affect with extraversion and negative affect with neuroticism that coincide with another research (Watson and Pennebaker, 1989; Clark et al., 1994). This relationship is explained by a high conceptual congruence evidenced in some definitions of temperament (Clark and Watson, 2008), which positions extraversion and neuroticism as two factors linked to affectivity from a genetic basis (Bouchard and Loehlin, 2001) and biological substrates (Depue and Collins, 1999). Thus, neuroticism and extraversion are strongly associated with individual differences in affective experience (Watson et al., 1988; Meyer and Shack, 1989), which in turn allows evidence of systematic links between the neurobiological bases of mood, emotions, and temperament (Clark and Watson, 2008). Additionally, there are no collinearity problems from the VIF values ( PA=1.153;NA=1.137) . Additionally, there is a conceptual similarity between neuroticism with NA, and extraversion with PA, which is observed in the moderate correlations that we found between these variables.

On the other hand, it has been shown that cognitive ability, measured through cognitive aptitude, often has a weaker relationship with academic success in samples of university students (Furnham et al., 2003), as evidenced by the decrease in the variability of scores in summative evaluations (Furnham et al., 2003). One explanation could be that the operationalization of the academic performance variable has been changing (Ackerman et al., 2001), for example, at the university level, greater emphasis has been given to other factors to evaluate performance, such as participation in the classroom (Furnham et al., 2003). Likewise, another likely reason is that there is some scope restriction at the university level as people with less cognitive ability are less likely to apply or be accepted into university courses. Guzmán and Serrano (2011) indicated that the applicants most likely to enter had studied in private primary schools with excellent conditions, had obtained high averages at the time of graduation and had access to cultural and educational resources. Therefore, personality traits could be considered a psychological variable of relevance in predicting academic performance (Ackerman et al., 2001; Furnham et al., 2003), for example, in the relationships between personality traits and the grades obtained in mathematics (Zhang and Ziegler, 2018). Our results show relationships between personality traits and academic performance, which coincides with other research where links between these variables were studied, justified by the importance of perseverance and responsibility in study (Rothstein et al., 1994). Just as a person’s aptitudes reflect what he or she can do, personality traits reflect how he or she can do it (Furnham et al., 2003). Thus, academic performance may be better predicted by obtaining a behavioral profile from personality trait measure than from intelligence tests (Goff and Ackerman, 1992). Thus, the personality measure used in this research has been considered as one of the most comprehensive and promising explanations for the measurement of personality (McAdams and Donnellan, 2009).

The personality traits extraversion and conscientiousness also presented a direct and statistically significant influence on performance, but in a negative and positive way, respectively. In addition, the personality trait extraversion had an indirect impact on academic performance, through its statistically significant and positive influence on the positive affect dimension. This is interesting because methodological strategies could be developed in the classroom that consider these personality traits. Openness to experience, characterized by creative behaviors in unknown contexts (Ackerman and Heggestad, 1997), did not present a statistically significant relationship with academic performance in mathematics. We believe that the characteristics of engineering students, mainly styles of approaching study in a concrete, structured and practical way (Bitran et al., 2004) would serve to understand this result. In an exploratory manner, we found that extraversion in women has a negative relationship with academic performance in mathematics, that is, less extroverted women present higher results.

This is interesting because from socio-epistemological theory, feminist theory, and the theory of social representations, it is evident that girls and women see the possibility of participating in the construction of mathematical knowledge as limited (Simón-Ramos et al., 2022). Thus, it is not surprising that in careers related to mathematics the enrollment of women is 30% (Simón, 2018), evidencing the exclusion that women experience, denying their epistemic authority, making their activities, interests and denigrating their styles of knowledge invisible (Blázquez, 2012).

Along these lines, differences have been reported in mathematical performance between female and male students of different levels (Eccles, 1989), explained by beliefs and conceptions (Andrews and Hatch, 2000), motivation (Middleton and Spanias, 1999) and attitude toward mathematics (McGraw et al., 2006). For the socio-epistemological theory of educational mathematics, the problem focuses on the democratization of learning (Cantoral et al., 2014), since in the traditional classroom, women, and other social groups (ethnic minorities, low-income, disability conditions, etc.) are excluded from the construction of mathematical knowledge for reasons unrelated to their abilities in mathematics.

Regarding cooperation, there is a statistically significant association of certain personality traits such as extraversion, agreeableness, and neuroticism, but they fail to indirectly influence academic performance, since cooperation does not have a statistically significant effect on this variable. When undergraduate and school students who made up the study sample had to decide how much money to give (in the economic game in which they participated), without being certain of receiving money from their peers, or of the amount they would receive, they were faced with the decision to cooperate (contribute money) or not, assuming the possible cost of being left without money in their favor. This was relevant because both educational establishments declare their educational model to be student-centered, where collaborative work is fundamental in their teaching systems.

These results could be understood from the existing risk aversion to a decision that implies an economic cost (Aguado, 2023). Thus, there is an instance in which those who make the decision to cooperate tend not to give importance to the absence of economic retribution from some players because they trust that they will also receive it from some player (Aguado, 2023). The non-existence of an association between cooperation and grade in mathematics could be explained by the characteristics of the graduation profile of a career such as commercial engineering, which the university students in the sample were studying. There are antecedents that propose that graduates of this career should evidence the ability to carry out entrepreneurial projects focused on maximizing their profits, where a high level of individualism is consistent with the required entrepreneurial attitude, but a high level of collectivism, even if it increases the social benefit, does not influence the maximization of profits (Felipe, 2016). Thus, students in this type of careers show attitudes based on the importance of numerical and purely quantitative elements over instances of reflection that incorporate social and personal dimensions (Argandoña et al., 2018). Additionally, the relationship between cooperation and achievement can be explained by aggressive behaviors among peers as mediating variables, such as bullying. Indeed, personality traits have been found to be associated with bullying (e.g., van Geel et al., 2017; Tatiani, 2023), which in turn may be related to different levels of cooperation, to explain their effect on academic performance. For example, Mitsopoulou and Giovazolias (2015) conducted a systematic review and meta-analysis showing that lower levels of agreeableness and conscientiousness and higher levels of neuroticism and extraversion are related to bullying as a victim and perpetrator in children and adolescents, which can be considered for future studies. It is expected that the results presented here constitute a contribution to future studies on strategies to improve student support in mathematics education contexts, incorporating affective, socio-behavioral dimensions and personality traits. In this way, the influence of these dimensions on mathematics students must be considered in educational practices to move towards scenarios conducive to learning.

### Projections

The results obtained in our research allow us to project future studies that consider other curricular programs based on the theoretical-conceptual model proposed here, a larger sample size, explore other techniques such as bootstrapping, adjust some variables that may present collinearity, and also to incorporate other measures associated with prosociality, such as aggression. We also hope to perform a comparison of models with and without indirect effects or employ standard model building/trimming strategies.

## Data availability statement

The original contributions presented in the study are included in the article/supplementary material, further inquiries can be directed to the corresponding author.

## Ethics statement

The studies involving humans were approved by Ethics Committee of The University of Santiago de Chile code 258. The studies were conducted in accordance with the local legislation and institutional requirements. Written informed consent for participation in this study was provided by the participants’ legal guardians/next of kin.

## Author contributions

FM-A: Conceptualization, Data curation, Formal analysis, Investigation, Methodology, Supervision, Writing – original draft, Writing – review & editing. LF-P: Conceptualization, Methodology, Supervision, Writing – review & editing. JM-R: Formal analysis, Funding acquisition, Methodology, Project administration, Resources, Writing – review & editing. OF: Conceptualization, Data curation, Methodology, Writing – review & editing. PP: Formal Analysis, Writing – review & editing. JV: Investigation, Visualization, Writing – review & editing.
